# Multidisciplinary Approach to the Child with Sex Chromosomal Mosaicism Including a Y-Containing Cell Line

**DOI:** 10.3390/ijerph18030917

**Published:** 2021-01-21

**Authors:** Bauke Debo, Marlies Van Loocke, Katya De Groote, Els De Leenheer, Martine Cools

**Affiliations:** 1 Internal Medicine and Pediatrics, Ghent University and Department of Pediatrics, Division of Pediatric Endocrinology, Ghent University Hospital, 9000 Ghent, Belgium; bauke.debo@ugent.be (B.D.); marliesvanloocke@gmail.com (M.V.L.); 2 Internal Medicine and Pediatrics, Department of Pediatrics, Division of Pediatric Cardiology, Ghent University Hospital, Ghent University, 9000 Ghent, Belgium; Katya.DeGroote@UZGENT.be; 3 Department of Otorhinolaryngology, Ghent University Hospital, Ghent University, 9000 Ghent, Belgium; els.deleenheer@ugent.be

**Keywords:** sex chromosomal mosaicism, 45,X/46,XY mosaicism, Turner syndrome, growth hormone, germ cell cancer

## Abstract

Children born with sex chromosomal mosaicism including material derived from the Y chromosome may present with a broad phenotypical spectrum. Both boys and girls can present with Turner features and functional health problems typically associated with Turner syndrome, but the presence of Y-chromosomal material can modify some aspects of the condition. We retrospectively analyzed the results of our cohort of 21 individuals (14 boys, 7 girls) with sex chromosomal mosaicism including Y-derived material followed at Ghent University Hospital according to our local multidisciplinary Turner surveillance protocol. Results were compared with literature data, focusing on similarities and differences between girls and boys with this condition. Age at diagnosis was lower in boys compared to girls but the difference was not significant. Short stature is a key feature of the condition both in girls and boys, but skeletal maturation may be different between groups. The effects of growth-hormone therapy remain unclear. Cardiac (33%), ear-nose- throat (ENT) (77.8%) and renal (28.6%) problems were as prevalent in boys as in girls from our cohort, and did not differ from literature data. In line with literature reports, a significant difference in the presence of premalignant germ cell tumors between males (0%) and females (42.9%) was found (*p* = 0.026). Taken together, this study demonstrates the similarities between girls with Turner syndrome and children with sex chromosomal mosaicism including Y-derived material, regardless of the child’s gender. Nowadays, girls with Turner syndrome are offered a dedicated multidisciplinary follow-up in many centers. We advocate a similar follow-up program for all children who have sex chromosomal mosaicism that includes Y-derived material, with special attention to growth, cardiac and ear-nose-throat problems, gonadal function and malignancies.

## 1. Introduction

Sex chromosomal mosaicism is defined as the presence of two or more cell lines derived from the same stem cell line but with a different sex chromosomal constitution, presenting in one individual [1,2]. The condition occurs in approximately 1.5 out of 10,000 live births [3,4] and is characterized by a very broad phenotypical spectrum. If one of the cell lines contains material derived from the Y chromosome, specifically the testis-determining gene “*Sex determining Region on Y*” (*SRY*), the phenotype may range from females with Turner Syndrome to typical males, but may also include neonates with intermediate degrees of virilization. The latter group presents with atypical genitalia at birth [4,5,6].

The majority of cases originate from chromosome missegregation or anaphase lag during early embryonic mitosis. This induces two or three cell lines: 45,X, 46,XY and 47,XYY; however, the latter is often lost upon further development of the embryo. The diagnosis of sex chromosomal mosaicism can be made prenatally by chorionic villus sampling or amniocentesis [5,7]. Mosaicism diagnosed by prenatal fibroblast analysis should always be revised postnatally to determine the constitutional karyotype. Molecular karyotyping is part of the routine work-up in neonates who have neonatal lymphedema or ambiguous genitalia at birth [8]. In girls with 45,X monosomy, analysis of a second cell line such as from a buccal swab is advised, given that low-grade mosaicism is thought to underlie most, if not all, forms of live-born 45,X individuals [5,9].

Between 10 and 12% of all girls diagnosed with Turner Syndrome have sex chromosomal mosaicism that includes a cell line with a (partial) Y chromosome, here further referred to as “Y+ sex chromosomal mosaicism”. Males with this karyotype are excluded from this diagnosis (i.e., they should not be referred to as “male Turner”). Of all individuals who have Y+ sex chromosomal mosaicism, an estimated 95% will present with a typical male phenotype [3,9]. Both girls and boys with Y+ sex chromosomal mosaicism can develop typical Turner features (e.g., short stature, short 4th metacarpal or metatarsal, webbed neck or low set ears, which have all been related to the presence of the 45,X cell line). On the other hand, the presence of the Y chromosome in the 46,XY cell line can modify multiple aspects of the condition, most notably gonadal development and function, and hence the sexual phenotype [10]. It is well known that girls with Y+ sex chromosomal mosaicism may experience functional problems typically associated with Turner syndrome, such as poor growth, primary ovarian insufficiency (POI), cardiac, renal and ear-nose-throat (ENT) abnormalities, auto-immune thyroiditis, learning disability and behavioral problems. Therefore, these girls are enclosed in a multidisciplinary dedicated follow-up protocol, which has been recently updated [9]. If boys with this condition are indeed at risk for having these problems to the same extent, they should require the same surveillance program. However, applying this program in these boys has not been formally addressed so far [10].

To further explore this question, we analyzed data from the cohort of individuals (*n* = 21) followed at our center and reviewed the current literature. For the entire cohort, multidisciplinary longitudinal follow-up since diagnosis or referral to our center was organized according to the surveillance protocol as outlined in [9].

## 2. Patients and Methods 

This study was approved by our institutional ethical review board (reference B67020083610) and was conducted in accordance with the principles for clinical medical research as outlined in the declaration of Helsinki. 

### 2.1. Patients

For this retrospective, single-center study, the Ghent University hospital’s Disorders of Sex Development (DSD) database was searched between 2007 and 2020, identifying all individuals diagnosed with Y+ sex chromosomal mosaicism at our center or referred to our center after this diagnosis had been made elsewhere. A total of 21 individuals (14 males, 7 females) were included. Data were collected reviewing the medical files for information on genital phenotype at birth, age at diagnosis, cardiac, ENT, urinary and other organ abnormalities, growth including target height and growth hormone (GH) treatment, gonadal differentiation, hormone level (Luteinising Hormone (LH), Follicle Stimulating Hormone (FSH), Anti-Müllerian Hormone (AMH)) and puberty.

Blood samples were taken as part of routine clinical care. Laboratory tests were aimed at identifying abnormal liver tests, gluten enteropathy, auto-immune thyroiditis, insulin resistance and gonadal failure as well as for follow-up of potential growth hormone therapy. Abnormal liver tests were defined as the repetitive occurrence of liver values (serum glutamic oxaloacetic transaminase (SGOT), serum glutamic pyruvic transaminase (SGPT)) above the upper limit in three or more consecutive tests. The presence of auto-immune thyroiditis was verified by checking for anti-thyroglobulin and antithyroperoxidase antibodies in combination with thyroid function tests. 

The genital phenotype was described by the External Masculinization Score (EMS), or when phallic length was available by the newer External Genitalia Score [11,12]. Both scores were based on the genital aspect before eventual genital surgery took place. Growth and target height data were calculated based on the Flemish growth charts [13].

### 2.2. Statistical Analyses

Results are expressed as mean (range) for normally distributed continuous variables or as median (interquartile range (IQR)) otherwise. Standard deviations (SDs) of height parameters are based on the Flemish growth references [13]. Outcomes between males and females with Y+ sex chromosomal mosaicism were compared using a Fisher’s exact test, independent Student’s *t*-test (after verifying normal distribution by Shapiro–Wilk test), Welch modified *t*-test or Mann–Whitney U test as appropriate. For biochemical results, in cases with hormone levels below the limit of detection, this limit was input for analysis. Results were analyzed using SPSS software (version 25.0; Armonk, NY, USA: IBM Corp.) For all tests, the level of significance was set at 0.05.

### 2.3. Literature Review

The PubMed and Embase databases were searched using a combination of the search terms ‘45,X/46,XY mosaicism’, ‘disorder of sex development’ and ‘gonadal dysgenesis’. A selection of relevant articles was made based on title and abstract. Only full articles in English published from 1990 onwards were considered.

## 3. Results

### 3.1. Patient Cohort 

#### 3.1.1. Clinical Presentation 

Fourteen males and seven females who had been diagnosed with Y+ sex chromosomal mosaicism and who were followed at our institution were included. Patient characteristics are displayed in Table 1. Median age (IQR) at diagnosis was 0.1 years (0–0.7) in males and 5 years (0–14) in females. Phenotypical Turner features were found in 76.2% (16 out of 21) of all individuals. Short 4th metacarpal and/or metatarsal was the most consistent finding, occurring in six out of 21 (28.6%) patients.

The most frequent reasons for karyotyping in boys were aberrant prenatal tests (*n* = 6) and atypical genitalia at birth (*n* = 5). Two boys were investigated solely for short stature, and one for motor and mental delay. One boy with atypical genitalia (Table 2, patient ID 1) had a 46,XY karyotype in peripheral blood but the skin fibroblast and gonadal karyotype revealed 45,X/46,XY. In girls, karyotyping was done for the investigation of short stature (*n* = 3) or delayed puberal onset (*n* = 1). The remaining three girls were tested in the neonatal period due to atypical genitalia at birth. The EGS could not be calculated in one girl due to missing data, the two others had an EGS of 4 and 5. 

Genetic data are represented in Table 2. Marker chromosomes were identified as derived from the Y chromosome by fluorescent in situ hybridization (FISH), and presence of *SRY* was confirmed in all cases, including in girls with atypical genitalia (cases 16, 17 and 18).

#### 3.1.2. Growth

Mean height SD at first visit was −0.91 in boys and −2.33 in girls. This was −0.95 and −1.84 SD below midparental height in boys and girls, respectively.

In our study cohort, 14 individuals (eight males and six females) had received or were receiving growth hormone (GH) therapy (50 µg/kg/d) at the time of data collection, whereas four males and one female were considered too young (i.e., below 4 years of age) for such therapy. One boy was currently 6 years old and growing according to his genetic potential. The remaining male was diagnosed at the end of puberty. In case 9, a deletion of the pseudoautosomal region 1 (PAR1) on the Y chromosome, including the *Short Stature Homeobox* (*SHOX)* gene, was found, further compromising growth expectations. GH had been started at a mean age of 11.5 years in girls and 8.8 years in boys (ns). At the start of GH, mean height SD was −2.96 in girls and −2.23 in boys (2.5 and 2 SD below target height, respectively), whereas chronological age–bone age, further referred to as ∆ bone age was +2 years in girls and −1 year in boys. After 1 year of therapy, mean ∆ height SD was 0.76 in girls and 0.67 in boys (ns).

At the time of data collection, four males and three females had reached a median final height of −1.8 SD. This was 2.2 SD below midparental height. Six of them had been treated with GH for a mean (SD) duration of 57.2 months (21.7).

#### 3.1.3. Cardiac Anomalies

Our institutional cardiac surveillance protocol [14] was applied in all individuals with Y+ sex chromosomal mosaicism. Cardiac anomalies were identified in seven children (33.3%), with a predominance of left-sided heart defects. No children were diagnosed with hypertension. There were no differences in the frequency or nature of identified cardiac anomalies between males and females. Findings are detailed in Table 1 and Table 3.

#### 3.1.4. Biochemical, Renal and ENT Findings

A structural renal ultrasound was performed in all patients at diagnosis. Liver function tests, auto-immune thyroid screening and screening for gluten enteropathy were performed yearly. ENT screening, including hearing tests, were performed in 85.7% (18 out of 21) of participants. Individual follow-up was organized according to initial findings. Taken together, ENT problems were identified in 14 out of 18 (77.8%) individuals, with no differences in ENT issues between female and male individuals with 45,X/46,XY. A significant number of children had a history of recurrent acute otitis media (AOM) (55.6%). Hearing screening in 18 out of 21 children indicated normal hearing in 83.3%, with 3 out of 18 displaying conductive (*n* = 2) or sensorineural (*n* = 1) hearing loss. Detailed results are presented in Table 1 and Table 3. In six (28.6%) children, all boys, renal anomalies were identified. One boy and one girl had three consecutive measurements of liver values exceeding the upper limit. One girl tested positive for antithyroperoxidase antibodies with normal thyroid hormone levels. There was no evidence of gluten enteropathy in the 17 tested children.

#### 3.1.5. Gonadal Function and Germ Cell Cancer (GCC) Risk

In girls, there was no correlation between the genital phenotype or the gonadal differentiation pattern and the distribution of the cell lines in peripheral blood lymphocytes (Table 2 and Table 4). Six females underwent bilateral gonadectomy after diagnosis, in view of the increased GCC risk and poor perspectives of gonadal function [25]. Gonadectomy is planned in the seventh girl, currently 4 years old. In situ gonadoblastoma lesions were found in three girls, but no invasive dysgerminoma was seen. Two girls with bilateral gonadoblastomas had a typical female phenotype and had been diagnosed due to short stature. One girl with unilateral gonadoblastoma had atypical genitalia at birth.

Seven males underwent gonadectomy (two bilateral) in early childhood, mostly due to the presence of dysplastic abdominal gonads that could not be descended into the scrotum. No invasive GCC or premalignant lesions were encountered (Table 4). Males had significantly less GCC lesions as compared to females in our study cohort (*p* = 0.026). Four out of 12 boys who had at least one gonad entered puberty spontaneously (patient 1, 3, 5 and 6), while the remaining eight had no signs of pubertal onset yet but also no elevated gonadotropin levels so far. Sperm analysis was performed in two young adult men (patients 1 and 5) revealing azoospermia. Germ cells were seen in a testicular semen extraction (TESE) specimen of patient 1 who was diagnosed at birth with atypical genitalia. TESE revealed no germ cells in patient 5, who had typical male genitalia and was diagnosed due to short stature. Gonadal characteristics are presented in Table 4.

## 4. Discussion

### 4.1. Clinical Presentation and Karyotypes

Ninety-five percent of all individuals with Y+ sex chromosomal mosaicism have typical male genitalia. It is currently unknown how many of them attain medical attention (e.g., due to short stature or infertility). The advent of the non-invasive pregnancy test (NIPT) may shed light on this question in the coming years. In line with other studies [4,7,10], age at diagnosis in our cohort was much younger in children with atypical genitalia than in typical males or females and ranged from the prenatal period to adolescent age (Table 3). Moreover, reasons for karyotyping in our patients were in accordance with literature data and included aberrant prenatal testing, atypical genitalia at birth, motor and mental delay, growth failure and unexplained pubertal delay. In adults, infertility is an additional reason for karyotyping [17].

It has been found that genotype–phenotype correlations in individuals who have Y+ sex chromosomal mosaicism are extremely difficult to detect even in the largest series due to heterogeneity in cell lines, the extent of mosaicism in various tissues, patient ages and diverse phenotypical descriptions [9], which is confirmed in our case series. Importantly, phenotypical Turner features were seen in 10 out of 14 boys (71.4%) and six out of seven girls (85.7%) (ns). The reported prevalence of Turner features in children who have Y+ sex chromosomal mosaicism varies from 14 to 70%, with a higher prevalence overall in girls as compared to boys with this karyotype. However, this may be due to reporting bias, as the majority of boys with typical male genitalia will remain undiagnosed [3,7,10,18,19,20]. The presence of minor genital anomalies (unilateral cryptorchidism, glandular hypospadias, bilateral testicular hypotrophy or asymmetry in testicular volume) does not seem to correlate with the presence of Turner features in boys [21]. Huang et al. observed cubitus valgus (72%), short stature (68%) and webbed neck (50%) as the most consistent Turner features in children with Y+ sex chromosomal mosaicism [16]. Our observation of the short 4th metacarpal/metatarsal as the most consistent finding (four out of 10 boys with Turner features (40%)) in boys from our cohort has not been observed in other studies but merits further attention, as it can increase awareness and guide clinicians towards karyotype testing (e.g., in males with typical genitalia who consult for short stature).

### 4.2. Growth

Short stature is a common finding in children or adolescents with Y+ sex chromosomal mosaicism. Karyotyping is routinely done in females with unexplained short stature, regardless of the presence of Turner features, but this may be to a lesser extent the case in boys presenting with unexplained short stature. Moreover, as over 90% of individuals with Y+ sex chromosomal mosaicism have a typical male phenotype, it is expected that most will not come to medical attention. These two factors complicate estimating the burden of growth problems in boys with Y+ sex chromosomal mosaicism [3,9,18].

According to a recent Chinese study, height started to decline to a median SD of −2.6 after the age of 2 years, with no significant differences between boys and girls [15]. In this study, other clinical signs were mostly absent. Also Bertelloni et al. recommend karyotyping in all boys with unexplained short stature [22].

Although growth hormone therapy is frequently used in children with Y+ sex chromosomal mosaicism, clinical evidence for this treatment is scarce. Some studies have reported that the response to short term GH treatment in boys who have sex chromosomal mosaicism is similar to what is observed in girls with Turner syndrome. It was also found that although GH therapy can improve short term growth, final height is often disappointing [4,16,18,20]. Other studies show no significant height gain in treated versus untreated boys [7]. Of note, mean ∆ bone age (chronological age-bone age) at the start of GH therapy in our cohort was significantly (*p* < 0.001) different in boys as compared to girls (−1.29 in boys, 1.80 in girls), which merits attention in further studies, as this may substantially underestimate the impact of GH treatment in boys as compared to girls with this condition.

### 4.3. Cardiac Anomalies

Cardiovascular anomalies are the major cause of excess mortality in women who have Turner syndrome. Approximately 25–50% of all Turner patients have structural heart defects. Hypertension and left heart defects such as coarctation aortae, aortic dilation or aneurysm and bicuspid aortic valve are most commonly seen [9,14]. Similar cardiovascular anomalies have been described in males and females with Y+ sex chromosomal mosaicism; however, the extent to which they are present in this population has been poorly documented. In our cohort, 33.3% of all children had cardiac anomalies with no difference between boys and girls, which is in line with the literature data (Table 3) [4,7,10,14,16,19,21]. Although none of the children in our cohort had been diagnosed with hypertension so far, we believe that 24 h ambulatory blood pressure monitoring should remain included in standard surveillance protocols given the many similarities of other cardiovascular findings with Turner Syndrome in this population [9,14].

### 4.4. Biochemical, Renal and ENT Findings

Up to 25% of all women with Turner syndrome may have renal anomalies such as horseshoe kidney, abnormal positioning or duplication of renal ureters or vessels and renal aplasia [9]. Available evidence suggests similar rates of renal anomalies (11–31%) in males and females with Y+ sex chromosomal mosaicism [1,10,16]. However, none of the girls from our cohort had renal anomalies, as compared to almost half of the boys; it is currently unclear what underlies this difference.

Few data exist on the prevalence of ENT problems in individuals who have Y+ sex chromosomal mosaicism. ENT problems were very frequent in our cohort and occurred as often in boys as in girls (Table 3). We therefore believe that ENT surveillance in childhood is equally important for both boys and girls with this condition, as it is for girls with Turner syndrome. According to Gravholt et al., up to 66% of Turner syndrome patients suffer from middle ear disease. Conductive hearing loss occurs in 25–40% of girls with Turner syndrome, typically in child- and adulthood. It is associated with persistent secretory otitis media, chronic otitis media, pars flaccida retraction pocket and cholesteatoma. Sensorineural hearing loss (SNHL) can occur as early as 6 years of age. In the age group of 11- to 20-year-old girls, 11% suffer from SNHL and up to one-third of all Turner girls overall have some degree of SNHL [9,26]. No data are currently available on the prevalence of conductive hearing loss in individuals who have Y+ sex chromosomal mosaicism. Given the impact of hearing on quality of life, we strongly suggest further research on this topic and systematic hearing screening of all individuals with Y+ sex chromosomal mosaicism.

Autoimmune conditions such as pernicious anemia, Hashimoto thyroiditis, anti-adrenal and anti-GAD (glutamic acid decarboxylase) autoantibodies have been equally described in boys and girls who have Y+ sex chromosomal mosaicism [21]. However, the only autoimmune condition found in our study cohort was the presence of anti-TPO antibodies in one female. This is in line with the 0–22% thyroiditis and hypothyroidism found in other series and is likely due to the young median age of our cohort [4,8,10,16,21].

### 4.5. Gonadal Function and GCC Risk

In our series, no correlation was found between the extent of mosaicism for the Y-containing cell line in peripheral blood lymphocytes and the genital or gonadal phenotypes. Presence of the testis-determining gene *SRY* was confirmed by FISH in all cases with a structurally abnormal Y chromosome. It was shown previously that even mosaic karyotypes at the gonadal level do not correlate with gonadal differentiation patterns, suggesting that timing and spatial thresholds of SRY signaling are equally important in determining the fate of the gonad, next to the number of Y material-containing cells [27].

Optimal management of gonads in children with Y+ sex chromosomal mosaicism requires weighing the risk for GCC development against the benefits of hormone and eventually fertility preservation on a case-by-case basis [25]. In none of the seven girls from our cohort was any functional ovarian tissue found. Bilateral gonadoblastomas were seen in two girls with typical female genitalia, questioning previous findings from our group [23], where in 48 cases with Y+ sex chromosomal mosaicism the highest GCC risk was seen in boys and girls with ambiguous genitalia and the lowest risk was in typical females. Therefore, previous recommendations to perform elective but early (i.e., prepubertal) gonadectomy in typical girls with sex chromosomal mosaicism is further supported by the current data [24,28].

The gonadal function, which is tightly related to gonadal histology, is highly variable in males and females with partial gonadal dysgenesis due to sex chromosomal mosaicism [23]. It was recently reported that males diagnosed based on the presence of atypical genitalia have lower rates of spontaneous puberty and higher rates of testosterone supplementation as compared to males diagnosed due to other reasons [4]. However, not all studies find such a correlation [7]. Generally speaking and in line with findings in our cohort, most boys with Y+ sex chromosomal mosaicism experience the spontaneous onset of puberty, progress through puberty at a normal pace and have normal testosterone levels, despite their genital anomalies and smaller testicular volumes. At the end of puberty, signs of impaired testicular function may be present (low testosterone and inhibin B, high FSH), and hormone replacement may be needed later in life. Azoospermia is often found in adulthood, supporting the suspicion of low or no fertility in males with Y+ sex chromosomal mosaicism, even in cases with typical male genitalia [4,7,17,21]. The use of advanced fertility preservation techniques such as TESE can offer opportunities in some males, as demonstrated in our cohort [29].

The co-presence and aberrant expression of testis-specific protein Y-encoded (TSPY) located on the short arm of the human Y-chromosome and the pluripotency factor Octamer Binding Protein 3 Transcription Factor 4 (OCT3/4) is hypothesized to increase the risk for malignant GCC development [30,31,32]. The risk of in situ neoplastic lesions has been estimated at 15–36.4% [24], but in the subgroup of children with ambiguous genitalia it may be as high as 55% [23]. Taking these data into consideration, and based on the positive prospects for endogenous testosterone production in many males with Y+ sex chromosomal mosaicism and partial testicular dysgenesis, it has been recommended to preserve scrotal gonads wherever possible and organize a strict surveillance program, including self-palpation, annual ultrasounds and a testicular biopsy at the end of puberty. Severely dysgenetic gonads that cannot be brought in a stable scrotal position can best be removed. In affirmed females with partial testicular dysgenesis, gonadectomy will prevent further virilization apart from GCC development [4,15,28,33].

## 5. Conclusions

Sex chromosomal mosaicism, including chromosome Y-derived material, is a highly variable condition that needs multidisciplinary and specialized care. Children and adults can present with a broad range of phenotypes and variable involvement of growth, cardiac, ENT and other organ anomalies, gonadal failure and GCC risk.

Analysis of our patient cohort revealed some important findings that have hitherto not been reported. The frequent observation of a short 4th metacarpal or metatarsal in children with Y+ sex chromosomal mosaicism could hint towards the diagnosis. This finding occurring in a male with a history of genital anomalies or short stature should prompt the clinician to request karyotyping. Skeletal maturation seems to be significantly different in girls and boys with Y+ sex chromosomal mosaicism and may substantially affect growth and response to GH therapy. The prevalence of growth problems, cardiac and renal anomalies and ENT-issues is similar in both girls and boys diagnosed with this condition. It is, however, important to keep in mind that the largest clinical group (i.e., males who have Y+ sex chromosomal mosaicism and a typical male phenotype) are mostly not diagnosed and therefore underrepresented in all studies. This selection bias makes it impossible to predict outcomes regarding growth, puberty, tumor risk, cardiac and other organ anomalies for this group of individuals with Y+ sex chromosomal mosaicism. Given the many similarities with Turner syndrome, the recently updated recommendations for the follow-up of girls with Turner syndrome may serve as a guideline for both males and females diagnosed with Y+ sex chromosomal mosaicism.

## Figures and Tables

**Table 1:** Patient characteristics about here.

Patient ID	Gender	Age at Diagnosis (y)	Current Age (y)	TS Features	Cardiac Anomalies	ENT Findings	Renal Findings	Abnormal Liver Tests *	Auto-Imm Unity	Other Health Issues	Height SD at Last Assessment	Duration GH (m) (∆CA-BA at Start (y))	∆ Height SD at 12 m GH	TH-SD-FH-SD
**MALES**														
1	M	0	21	Shield thorax, short 4th metatarsal, short arm span	VSD (spontaneously closed), bicuspid aortic valve, aortic dilation	NA	Horseshoe kidney, recurrent urinary tract infections	N	N	Orthopedic: lower limb length discrepancy	−1.5	89 (0)	0.7	−2.2
2	M	0	11	Shield thorax, multiple nevi		Recurrent AOM (VT placement) atelectasis, mild conductive hearing loss		N	N		0.4	(46) ° (−2)	0.5	
3	M	1.3	21	Shield thorax, short 4th metatarsal, short extremities, webbed neck	Bicuspid aortic valve, aorta ascendens dilatation, reduced LV function	Recurrent AOM (VT placement, adenotonsillectomy), eardrum retraction, mild conductive hearing loss		N	N	GERD, delayed neuromotor development, congenital fusion C2-C3, congenital stenosis cervical spinal canal	−1.8	67 (−1)	0.3	−2.2
4	M	0	11	Shield thorax, short extremities, webbed neck, wide spaced nipples	Bicuspid aortic valve (hypoplasia of the aortic arch, normalized with age)	Recurrent AOM (VT placement, adenotonsillectomy, bilateral otoplasty, nl hearing	Non-functional multicystic right kidney	N	N	GERD, bronchial hyperreactivity, volvulus, delayed psychomotor development	−0.8	(84) ° (−1)	0.9	
5	M	11	20	Short 4th metatarsal	Bicuspid aortic valve, aorta ascendens dilatation	Recurrent AOM (VT placement, adenotomy), nl hearing		N	N	ADHD, idiopathic scoliosis, phimosis, lower limb length discrepancy	−3.3	55 (−1)	0.5	−2.5
6	M	0	14	Muscled and short lower legs		NA		N	N	Enuresis nocturna + diurna	−0.9	(68) ° (−2)	0.7	
7	M	14	22	Short 4th metatarsal, short stature	Arteria lusoria	Nl hearing		Y	N		−3.3	-		−1.8
8	M	0	6			NA		N	N		−1.1	-		
9	M	0	14	Shield thorax, short lower arms, short and muscled lower legs		Nl hearing	Horseshoe kidney	N	N	precocious puberty, GERD	−1.8	(77) ° (NA)		
10	M	0	3			Recurrent AOM (VT placement, adenotomy), nl hearing	Left-sided hydronephrosis				−1.7	-		
11	M	0	3			Recurrent AOM and glue ear (VT placement, adenotomy), nl hearing				Delayed psychomotor development,	−0.3	-		
12	M	0	2			Nl hearing					0.8	-		
13	M	0	3	Lymfoedema hands and feet		Recurrent AOM and glue ear (VT placement, adenotomy), nl hearing	Horseshoe kidney	N		Multiple allergies, GERD, delayed speech development	−0.9	-		
14	M	6	13	Shield thorax, short extremities		Recurrent AOM (VT placement), nl hearing	Left-sided hydronephrosis	N	N		0.4	(30) ° (−2)	1.1	
**FEMALES**														
15	F	13	27	Shield thorax, short 4th and 5th metacarpal, naevi, webbed neck, cubitus valgus	Bicuspid aortic valve, aortic arch deformity	Eardrum retraction, mid and high-frequency sensorineural hearing loss, hearing aids		N	N	Hypercholesterolemia, vitamin D deficiency	−1.7	33 (3)	1.2	−1.4
16	F	0	19	Shield thorax, webbed neck, cubitus valgus, low posterior hairline	Patent ductus arteriosus, closed through catheterization-aorta ascendens dilatation	Adenotonsillectomy, nl hearing		N	Y		−2	72 (2)	0.2	−0.9
17	F	0	12	Short 4th metatarsal, short stature		Tonsillectomy, nl hearing		Y	N		−1.7	(50) ° (NA)	0.8	
18	F	0	4	Short stature, hypertelorism, wide nose bridge		Protruded ears, nl hearing		N	N		−3.4	-		
19	F	5	7	Short and muscled lower legs		Recurrent AOM, adenotonsillectomy, nl hearing		N	N	GERD	−1.4	(27) ° (0)	0.9	
20	F	14	17	Shield thorax, webbed neck, naevi, coxa valga		Recurrent AOM and glue ear, retraction (VT placement), nl hearing		N	N	Dyslexia, dyscalculia	−1.4	27 (2)	0.7	−2.4
21	F	14	15			Nl hearing		N	N	Bronchial hyperreactivity, GERD (Nissen procedure), retinal detachment, hypercholesterolemia, vitamin D deficiency, ADD, impaired glucose tolerance	−2.2	(14) ° (2)		

**Abbreviations**: AOM: acute otitis media, FH: final height, GH: growth hormone, LV: left ventricle, m: months, N: no, NA: not available, TH: target height, TS: Turner syndrome, VSD: ventricle septum defect, VT: ventilation tube, y: years, Y: yes. ∆CA-BA: difference between calendar age and bone age at start of GH-therapy, ∆ height at 12 m GH: height SD at 12 months GH-therapy-height SD at start GH-therapy. °: GH therapy ongoing; GERD: Gastro-Esophageal Reflux Disease. * abnormal liver tests are defined as repetitive occurrence of liver values (SGOT = serum glutamic oxaloacetic transaminase, SGPT = serum glutamic pyruvic transaminase) above the upper limit in 3 or more consecutive tests.

**Table 2:** Genetic results. All karyotypes were performed on peripheral blood lymphocytes, unless otherwise specified.

Patient ID	Karyotype	Method	SRY-Region
**MALES**			
1	46,XY arr(1-22)x2,(XY)x1 45,X (27%)/46,XY (73%) (skin fibroblasts) 45,X (67%)/46,XY (33%) (right gonad) 45,X (23%)/46,XY (77%) (left gonad)	Chromosome G-banding Array-CGH	
2	ish 45,X(19/100)/46,X,r(Y)(pterq10)(63/100)/46,X,dic r(Y)(pterq10)(18/100).ish r(Y)(pterq10)(Y97+,RP13-391G2+,RP11-1144P2-),dic r(Y)(pterq10)(Y97++,RP13-391G2++,RP11-1144P2-)	FISH	Present
3	45,X(9)/46,XY(14)	Chromosome G-banding	
4	45,X(6)/46,X,i(Y)(p10)(26) X(6)/X,i(Yp)(13) Arr Yp11.31p11.2(11-10622062)x3, arr Yq11.21q12(12571053-57440809)x1	Chromosome G-banding FISH Array-CGH	Present
5	45,X(74)/46,X,idic(Y)(p11.2)(39)/46,XY(11) X(71)/X,iso(Y)(37)/XY(11)	Chromosome G-banding FISH	Present
6	45,X(86)/46,XY(14) Arr Yq11.223q11.223(22492074-57441720)x0	Chromosome G-banding Array-CGH	
7	45,X/46,XY	Chromosome G-banding	
8	45,X(7)/46,XY(13) X(43)/XY(57)	Chromosome G-banding FISH	Present
9	45,X/46,Xidic(Y)(p11.3) Idic(Y)(p11.3)(839D20-)	Chromosome G-banding FISH, MLPA	Present
10	45,X,del(Y)(q11.2qter),der(13;14)(q10;q10)(75)/45,X,i(Y)(p10),der(13;14)(q10;q10)(25)	Chromosome G-banding	
11	45,X(6)/46,XY(4) X(51%)/XY(49%)	Chromosome G-banding FISH	Present
12	45,X,t(2;9)(q33.1;p13)(20/46,X,idic(Y)(q12),t(2;9)(q33.1;p13) (19) X(3)/X,idic(Y)(7)	Chromosome G-banding FISH	Present
13	45,X(9)/46,XY(8) X(107)/XY(115)	Chromosome G-banding FISH	Present
14	45,X(82)/46,X,Yq-(253) X(78)/X,Yq-(233)	Chromosome G-banding FISH	Present
**FEMALES**			
15	45,X(12)/46,XY(51) X(33)/XY(77)	Chromosome G-banding FISH	Present
16	45,X/46,XY/47,XYY X(192)/XY(5)/XYY(3)	Chromosome G-banding FISH	Present
17	45,X(5)/46,X,idic(Y)(q11)(6)/46,XY(4) X(3)/X,idic(Y)(q11)(4)/XY(1)	Chromosome G-banding FISH	Present
18	45,X(28)/46,X psu idic (Y) (q11) (15)/ 47,X psu idic (Y) (q11) + psu idic (Y) (q11) (7) psu Idic(Y) (SRY+)	Chromosome G-banding FISH	Present
19	45,X(4)/46,XY(2) X(63)XY(41)	Chromosome G-banding FISH	Present
20	45,X(3)/46,XY(3) X(37)XY(83)	Chromosome G-banding FISH	Present
21	45,X(5)/46,XY(24) X(35)/XY(175)	Chromosome G-banding FISH	Present

**Abbreviations**: CGH: comparative genomic hybridization, SRY: sex determining region on Y.

**Table 3:** Comparison of findings in girls and boys of this cohort with literature data.

	This Cohort		*p*-Value	Literature	References
	Male	Female			
**Clinical Characteristics**					
Gender	14/21 (66.7%)	7/21 (33.3%)		95% typical male phenotype, 4% male with genital anomalies, 1% female	[3,15]
Reasons for consultation	Aberrant prenatal tests (6/14-42.9%), atypical genitalia at birth (5/14-35.7%), short stature (2/14-14.3%), motor and mental delay (1/14-7.1%)	Short stature (3/7-42.9%), delayed puberal onset (1/7-14.3%), atypical genitalia at birth (3/7-42.9%)		Short stature (74%), atypical genitalia (37%), delayed puberty (5%)	[16]
Age at diagnosis (y)	0.1 (0–2.475)	5 (0–14)	0.173	Ranging from prenatal to adult age	[1,4,15,17,18,19]
TS features	10/14 (71.4%)	6/7 (85.7%)	0.624	M: 14–70% F: 44–89%	[3,7,15,16,18,19,20,21]
**Growth**					
Height at birth [mean (SD)]	−0.91 (1.01)	−2.33 (1.14)	0.010	50–67% IUGR , no difference between pre- or postnatal diagnosis, no influence on final height	[7,21,22]
FH SD [median (IQR)]	−2.5 (−3.3; −1.5)	−1.7 (−2; −1.4)	0.343	M: −2.2–−2.5 no significant difference in final height or pubertal growth spurt between patients with a prenatal and postnatal diagnosis, a minor abnormality of external genitalia is associated with a shorter adult height (ns when genetic potential was accounted for) F: >2 SD below the mean value for sex at the same age	[4,21]
Duration of GH therapy in (m) [mean (SD)]	70.3 (17.2)	44 (24.4)	0.028	7–154	[7]
Age (y) at start GH therapy [mean (SD)]	8.8 (2.33)	11.5 (4.32)	0.161	4.5–15.1	[7,16]
∆ height at 12 m GH [mean (SD)]	0.67 (0.27)	0.76 (0.36)	0.637	Median height ∆SDS gain (range) after 1 year [0.51 (0.1–1.2)]	[4]
**Organ anomalies**					
Cardiac anomalies	Structural heart defects 5/14 (35.7%)	Structural heart defects 2/7 (28.6%), hypertension 0%	>0.999	M: 0% to 26% F: 0% to 44% predominantly left-sided in both sexes (non-stenotic bicuspid aortic valve, aortic stenosis, coarctation)	[4,7,16,19,20,21]
Hypertension	0/14 (0%)	0/7 (0%)		M: 10% mild hypertension F: 25% mild hypertension	[9,14]
Renal anomalies	6/14 (42.9%)	0/7 (0%)	0.061	M: 11% to 20% no difference between boys who were diagnosed due to genital anomalies and boys diagnosed due to other reasons F: 11% to 31%	[4,7,15,19,20,21]
ENT problems	8/11 (72.7%); 8/11 (72.7%) recurrent AOM, 2/11 (18.1%) conductive hearing loss	6/7 (85.7%); 2/7 (28.6%) recurrent AOM, 1/7 (14.3%) sensorineural hearing loss	0.638	M: 6–29% recurrent AOM, 5% conductive hearing loss F: 29–67% recurrent AOM, 11% conductive hearing loss	[20,21]
Auto-immune conditions	0/14 (0%)	1/7 (14.3%)	0.333	M: 0–7.5% autoimmune conditions (pernicious anemia, Hashimoto thyroiditis, anti-adrenal and anti-GAD autoantibodies) F: 11–22% autoimmune thyroiditis	[16,19,20,21]
**Gonads**					
Tumor risk	0/14 (0%)	3/7 (42.9%) 1 bilateral and 2 unilateral gonadoblastoma	0.026	15–36.4%	[18,23,24]
Spontaneous puberty	4/5 (80%)	0/5 (0%)	0.048	M: 79.7% spontaneous puberty, significantly lower in group of males with genital anomalies.	[4,15]

**Abbreviations**: M: male, F: female, y: years, m: months, TS: Turner syndrome, IUGR: intra uterine growth restriction, IQR: interquartile range, AOM: acute otitis media, NA: not available, ∆ height at 12 m GH = height SD at start GH-therapy—height SD at 12 months GH-therapy, TH: target height, FH: final height, anti-GAD = anti-glutamaatdecarboxylase autoantibodies.

**Table 4:** Gonadal function and pathology.

Patient ID	Gonado-Tropins	HRT	Age at Gonadectomy (Years)	Side	Reason for Gonadectomy	Pathology	EMS	Spontaneous Puberty	Fertility Data
**MALES**									
1	Normal for age	N	0.75	R	Dysplastic abdominal gonad	Gonadal regression	7.5	Y	AMH nl, sperm -, TESE: some GC
2	Elevated	N	0.3	R + L	Left dysplastic abdominal gonad, right gonadal torsion	Bilateral dysgenetic testis, no germ cells	6		
3	NA	NA	NP	-			10	Y	AMH nl
4	Normal for age	N	1	L	Left dysplastic abdominal gonad, right gonadal torsion	Gonadal regression	9.5		AMH nl
5	Slightly elevated	N	NP	-			12	Y	AMH nl, sperm -, TESE: no GC
6	Normal for age	N	0.75	L	Left dysplastic abdominal gonad	Dysgenetic testis, no germ cells	8	Y	AMH nl
7	Normal for age	Y	3	R + L	NA	NA	3	N	HRT
8	Normal for age	N	NP	-			12		AMH ↓
9	Slightly elevated	N	1	R	Dysplastic abdominal gonad	Dysgenetic testis, no germ cells	AG		AMH ↓
10	NA	NA	NP	-			12		AMH nl
11	Normal for age	N	1	R	Dysplastic abdominal gonad	Streak	5.5		
12	Normal for age	N	NP	-			12		AMH nl
13	NA	N	NP	-			7		AMH ↓, TESE: immature tubuli, loss of GC
14	Normal for age	N	NP	-			9.5		AMH ↓, TESE: Sertoli cell only
**FEMALES**									
15	NA	Y	16	R + L	-	Bilateral streak gonads		N	HRT
16	Elevated	Y	1	R + L	-	NA		N	HRT
17	Elevated	Y	8	R + L	-	Left: dysgenetic testis with gonadoblastoma, right: streak		N	AMH nl
18	normal for age		NP	-					
19	NA	N	7	R + L	-	Bilateral undifferentiated gonadal tissue with bilateral gonadoblastoma			
20	Elevated	Y	15	R + L	-	Bilateral calcified gonadoblastoma		N	HRT
21	Elevated	Y	15	R + L	-	Right: streak gonad, left: gonadal regression		N	AMH ↓, HRT

**Abbreviations**: AG: ambiguous genitalia not further specified, AMH↓: at least one value under the reference range, EMS: External Masculinization Score, GC: germ cells, HRT: hormone replacement therapy, L: left, N: no, NA: not available, nl: normal, NP: not performed, R: right, sperm -: no sperm on sperm analysis, TESE: testicular sperm extraction, Y: yes.

## Data Availability

The data presented in this study are available on request from the corresponding author. The data are not publicly available due to privacy.
